# A multifocal IDH-mutant glioma with differential response to IDH inhibition: Role of quantitative neuroimaging

**DOI:** 10.1016/j.radcr.2026.01.096

**Published:** 2026-02-21

**Authors:** Omar Ibrahim, Nicholas S. Cho, Viên Lam Le, Francesco Sanvito, Jingwen Yao, Ashley Teraishi, Albert Lai, Phioanh L. Nghiemphu, Noriko Salamon, Fausto J. Rodriguez, Richard Everson, Timothy F. Cloughesy, Benjamin M. Ellingson

**Affiliations:** a UCLA Brain Tumor Imaging Laboratory (BTIL), Center for Computer Vision and Imaging Biomarkers, University of California, Los Angeles, CA, USA; b Department of Radiological Sciences, David Geffen School of Medicine, University of California, Los Angeles, CA, USA; c Department of Bioengineering, Henry Samueli School of Engineering and Applied Science, University of California, Los Angeles, CA, USA; d Medical Scientist Training Program, David Geffen School of Medicine, University of California, Los Angeles, CA, USA; e UCLA Neuro-Oncology Program, David Geffen School of Medicine, University of California, Los Angeles, CA, USA; f Department of Neurology, David Geffen School of Medicine, University of California, Los Angeles, CA, USA; g Department of Department of Pathology and Laboratory Medicine, David Geffen School of Medicine, University of California, Los Angeles, CA, USA; h Department of Neurosurgery, David Geffen School of Medicine, University of California, Los Angeles, CA, USA; i Department of Psychiatry and Biobehavioral Sciences, David Geffen School of Medicine, University of California, Los Angeles, CA, USA

**Keywords:** IDH-mutant oligodendroglioma, IDH-mutant astrocytoma, MRI, T2-FLAIR mismatch, Ivosidenib

## Abstract

We present a rare case of concurrent, spatially distinct 1p/19q-intact IDH-mutant astrocytoma and 1p/19q-codeleted IDH-mutant oligodendroglioma in the same patient with divergent responses to IDH inhibitor therapy. A 40-year-old male presented to the emergency room following a seizure and was found to have 2 distinct left hemisphere tumors. Both lesions underwent biopsy and resection, with updated molecular diagnosis 4 years later revealing IDH-mutant 1p/19q-intact astrocytoma and IDH-mutant 1p/19q-codeleted oligodendroglioma. Tumor volumes, T2-FLAIR mismatch percentage, and normalized apparent diffusion coefficient (nADC) were quantified from serial MRIs. At initial presentation, quantitative analysis revealed the posterior lesion (IDH-mutant 1p/19q-intact astrocytoma) exhibited 60.6% T2-FLAIR mismatch volume and elevated median nADC of 2.72, while the anterior lesion (IDH-mutant 1p/19q-codeleted oligodendroglioma) showed minimal T2-FLAIR mismatch (1.8%) and lower median nADC (1.94). Volumetric analysis during IDH inhibitor (ivosidenib) therapy revealed differential response: the oligodendroglioma decreased in volume by 32.6% over 19 months, while the astrocytoma demonstrated progressive growth with a 77.3% volume increase over 19 months. This case demonstrates the utility of quantitative neuroimaging for accurate characterization of concurrent IDH-mutant gliomas with distinct molecular profiles. The observed differential response to IDH inhibition supports emerging evidence of molecular subtype-specific treatment sensitivities in IDH-mutant gliomas.

## Introduction

Molecular classification of gliomas has revolutionized our understanding of these tumors, with isocitrate dehydrogenase (IDH) mutation status and 1p/19q codeletion serving as critical markers for diagnostic precision since the 2016 World Health Organization Classification of Central Nervous System Tumors [1,2]. While the literature extensively covers the imaging characteristics and treatment response of single lesions, there is limited information on the rare presentation of concurrent gliomas with distinct molecular profiles in a single patient. To our knowledge, this phenomenon has only been described in 2 case reports involving 3 patients [3,4].

We present an exceptional case of a patient with 2 spatially distinct IDH-mutant gliomas in the same hemisphere—one with 1p/19q-codeletion (oligodendroglioma) and one without (astrocytoma)—highlighting both the value of advanced quantitative neuroimaging techniques in molecular diagnosis and the differential response of these lesions to IDH inhibitor therapy.

## Case presentation

### Clinical presentation and initial evaluation

A 40-year-old male with no significant medical history presented to an emergency department following a witnessed seizure. His symptoms had gradually evolved over 6 months, with episodes of confusion, aphasia, headaches, right peripheral visual field loss, and right-sided apraxia. His occupational history was notable for 18 years of aluminum exposure with previously documented elevated blood levels of heavy metals.

Initial brain MRI revealed 2 distinct lesions: a contrast-enhancing lesion in the left parieto-temporal region and a non-enhancing lesion in the left frontal lobe. The patient underwent biopsy of both lesions at an outside hospital, followed by craniotomy for resection of both tumors 2 weeks later. Initial histopathological diagnosis classified the left frontal tumor as a grade 3 astrocytoma and the left parieto-temporal tumor as glioblastoma (per pre-2021 WHO classification criteria [1]). After resection, tissue sampling for molecular diagnosis was only performed on the parieto-temporal lesion, and molecular diagnosis was grade 3 IDH-mutant 1p/19q-intact astrocytoma.

### Quantitative imaging analysis suggests different molecular diagnoses for different lesions

**Fig. 1:**
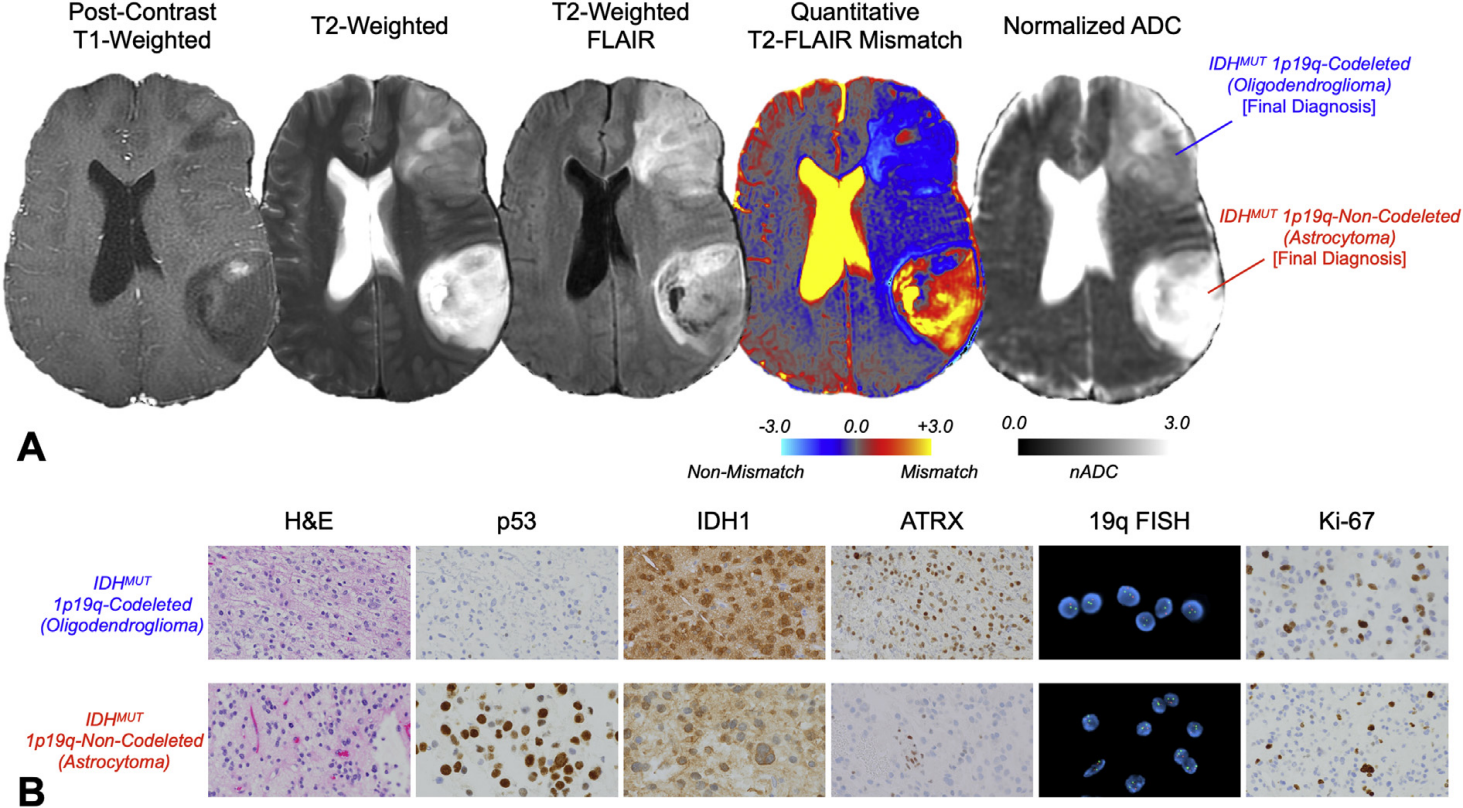
Multiparametric MRI characteristics at baseline and pathological data for a 40-year-old male with multiple suspected lower grade gliomas. (A) Axial images demonstrate distinct radiographic features of the parieto-temporal IDH-mutant 1p/19q-intact astrocytoma (IDHm-A) and frontal IDH-mutant 1p/19q-codeleted oligodendroglioma (IDHm-O). The parieto-temporal lesion exhibited pronounced T2-FLAIR mismatch (60.6% of tumor volume) and markedly elevated normalized apparent diffusion coefficient (nADC = 2.72), while the frontal lesion showed minimal T2-FLAIR mismatch (1.8%) and moderate nADC elevation (1.94), reflecting potentially distinct molecular profiles despite shared IDH mutation status. (B) IHC and FISH images from the second resection (at our institution) showing H&E, p53, IDH1, ATRX, 19q FISH, and Ki-67 results.

When retrospectively analyzed using quantitative neuroimaging techniques, the baseline MRI revealed distinctive features that challenged the initial histopathological diagnosis (Fig. 1A). The posterior lesion demonstrated striking T2-weighted hyperintensity with relative FLAIR hypointensity—a pattern consistent with the ``T2-FLAIR mismatch sign'' [[5], [6], [7]]. Quantitative analysis confirmed this observation, showing that 60.6% of the tumor volume exhibited T2-FLAIR mismatch [8]. Additionally, the normalized apparent diffusion coefficient (nADC) was markedly elevated at 2.72, consistent with features typically observed in IDH-mutant, 1p/19q-intact astrocytomas [9,10].

In contrast, the anterior lesion showed minimal T2-FLAIR mismatch (only 1.8% of tumor volume) and a moderately elevated nADC of 1.94. These quantitative metrics were more consistent with an IDH-mutant, 1p/19q-codeleted oligodendroglioma than an astrocytoma, suggesting a potential misdiagnosis of the frontal lesion [9].

### Delayed treatment and updated molecular diagnosis

Following the initial surgery, the patient declined standard chemoradiation and instead pursued various alternative treatments. After nearly 4 years of gradual tumor progression, the patient presented to our institution with worsening word-finding difficulty. Pre-surgical imaging at 45 months post-diagnosis showed increasing mass effect and continued growth of both lesions, with persistent T2-hyperintensity and re-development of relative FLAIR hypointensity in the parieto-temporal lesion compared to the frontal lesion.

Subsequent resection at our institution led to an updated molecular diagnosis: the left frontal lesion was reclassified as a grade 3 IDH-mutant 1p/19q-codeleted oligodendroglioma, while the left parieto-temporal lesion was confirmed as a grade 3 IDH-mutant 1p/19q-intact astrocytoma. This molecular confirmation aligned with the quantitative imaging features observed at baseline, demonstrating the potential value of advanced MRI techniques over simple needle biopsy in terms of accuracy of molecular diagnoses (Fig. 1B).

## Differential response to IDH inhibitor therapy

### Treatment course

Given the patient's confirmed IDH-mutant diagnoses and continued reluctance to undergo standard radiation and temozolomide therapy, treatment with the IDH inhibitor ivosidenib (Tibsovo, Servier Pharmaceuticals, Boston, MA) was initiated 47 months after initial diagnosis. Due to subjective complaints of palpitations after the first dose of the standard 500 mg twice daily regimen, the dosage was reduced to 250 mg twice daily, which the patient took intermittently for approximately 19 months. During this treatment period, the patient also received various alternative therapies at another institution, including atorvastatin, metformin, mebendazole, and doxycycline.

### Divergent tumor response

**Fig. 2:**
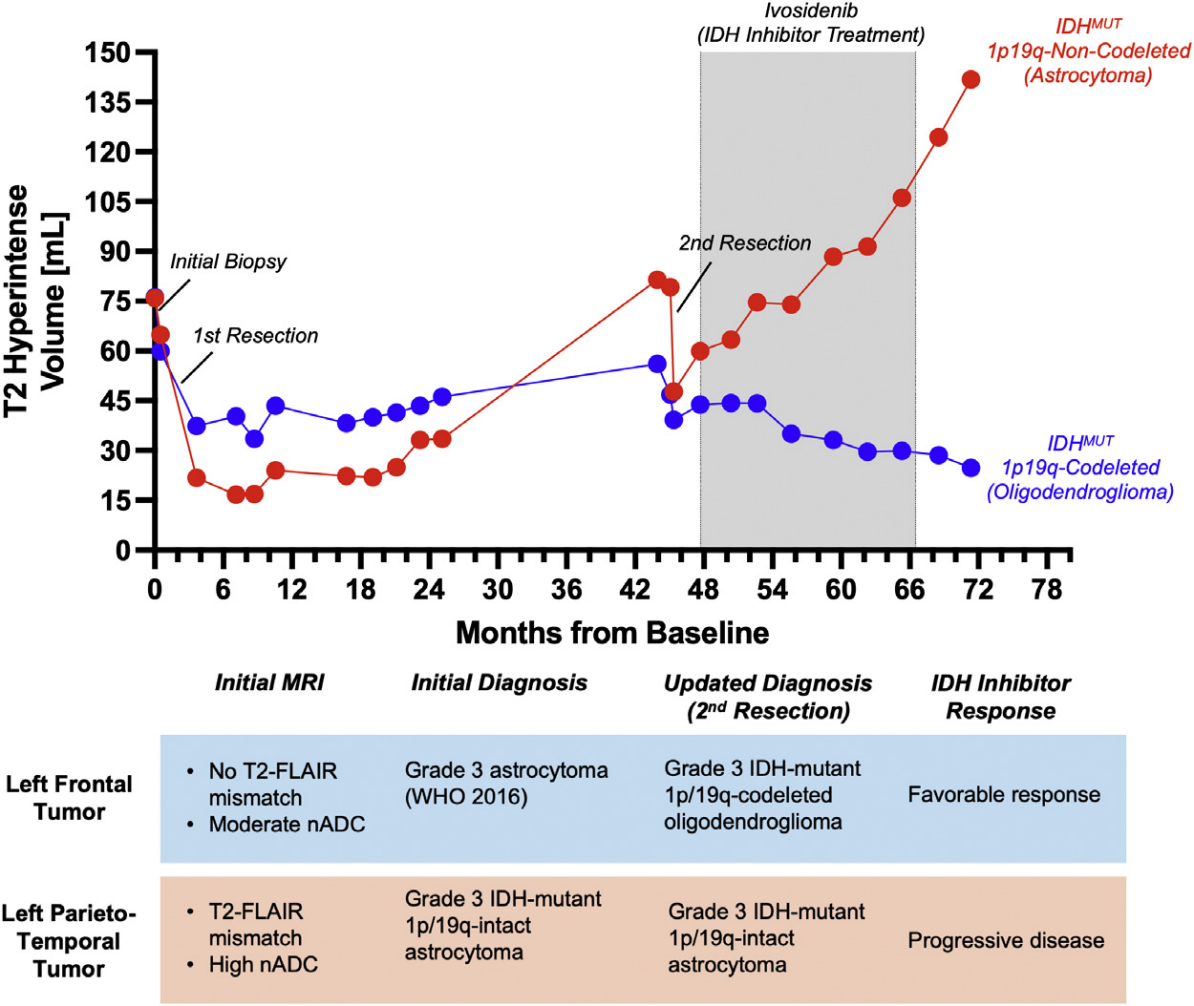
Longitudinal volumetric analysis of the 2 IDH-mutant lesions over 72 months of evaluation. Whole T2 hyperintense tumor volumes (mL) (including areas of contrast enhancement) were plotted against time from baseline (months) for both the IDH-mutant 1p/19q-intact astrocytoma (red) and IDH-mutant 1p/19q-codeleted oligodendroglioma (blue) using guidance from NS-HGlio [15] (Neosoma Ing, Groton, MA, neosomainc.com). Key clinical interventions and therapeutic regimens are annotated, highlighting the divergent response to ivosidenib treatment (47-66 months): 32.6% volume reduction in the oligodendroglioma versus 77.3% volume increase in the astrocytoma, demonstrating molecular subtype-specific sensitivity to IDH inhibition.

**Fig. 3:**
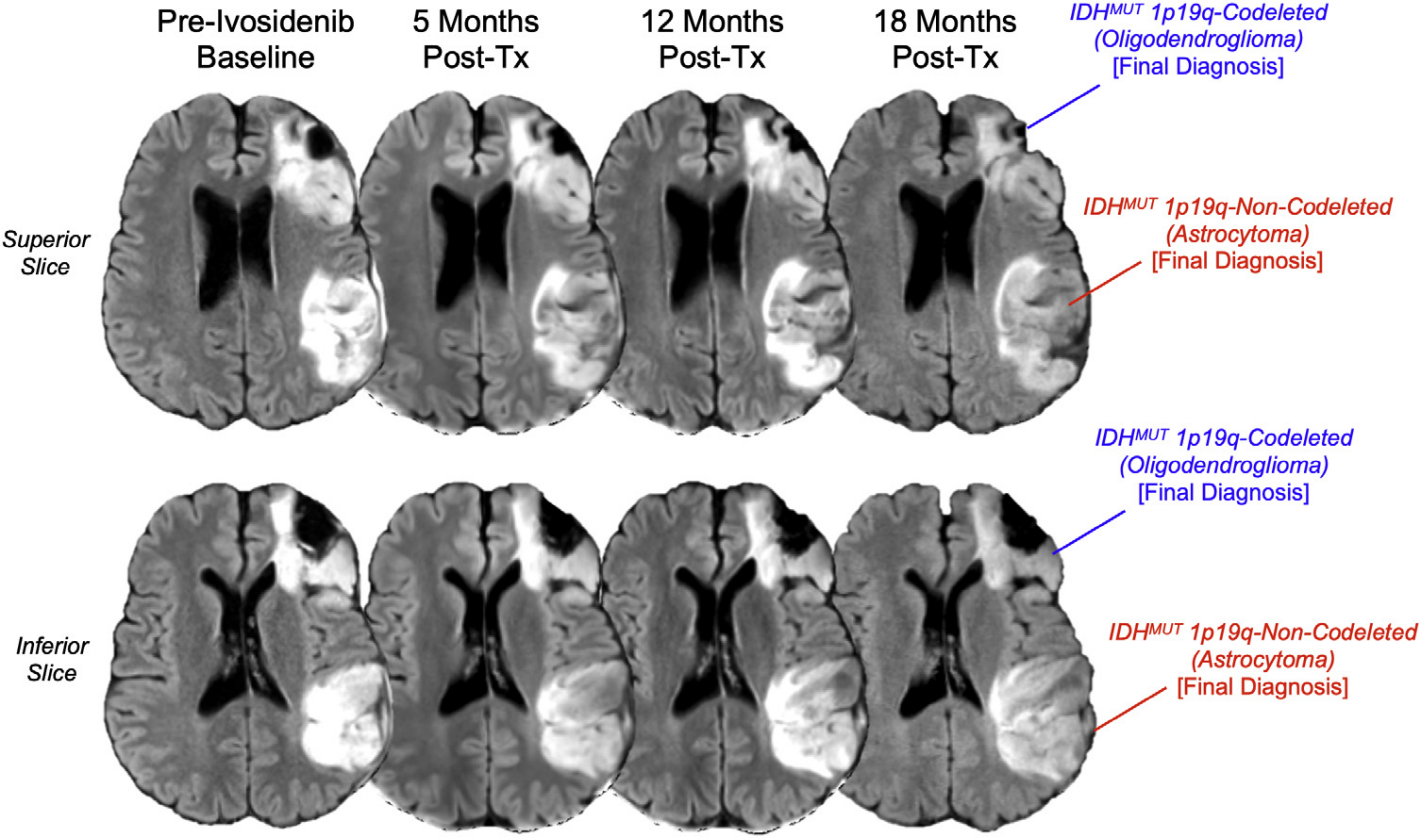
Serial normalized T2-weighted FLAIR images demonstrating differential response to IDH inhibitor therapy. Axial images acquired during the 19-month course of intermittent ivosidenib treatment (250mg BID) show progressive enlargement of the parieto-temporal IDH-mutant 1p/19q-intact astrocytoma with persistent T2-FLAIR mismatch characteristics, contrasted with gradual volume reduction of the frontal IDH-mutant 1p/19q-codeleted oligodendroglioma, illustrating subtype-specific treatment effects within the same patient.

Volumetric analysis [15] over the course of ivosidenib treatment revealed a striking differential response between the 2 lesions (Fig. 2, Fig. 3). The IDH-mutant 1p/19q-codeleted oligodendroglioma demonstrated a favorable response, decreasing in volume from 43.9 mL at treatment initiation to 29.6 mL by the end of the treatment period—representing a 32.6% reduction in tumor volume. In stark contrast, the IDH-mutant 1p/19q-intact astrocytoma continued to grow despite IDH inhibition, increasing from 59.9 mL to 106.2 mL during the same period—a 77.3% increase in volume. This divergent response is particularly notable as both tumors had exhibited growth prior to the initiation of ivosidenib therapy.

### Subsequent clinical course

Following discontinuation of ivosidenib due to subjective palpitations, the patient was treated with various other regimens, including dasatinib, pazopanib, and everolimus, all of which were discontinued due to side effects. He later received bevacizumab for 3 months, followed by repeat resection of the progressively symptomatic IDH-mutant astrocytoma. The patient ultimately began standard temozolomide and radiation therapy 81 months after initial diagnosis.

## Discussion

### Value of quantitative imaging in molecular diagnosis

The initial misdiagnosis of both lesions highlights the limitations of biopsies and histopathology alone, particularly when not accompanied by comprehensive molecular testing. Quantitative MRI parameters at baseline—specifically volumetric T2-FLAIR mismatch percentage and nADC values—correctly suggested the molecular classifications that were confirmed years later. This case supports the growing body of evidence that quantitative imaging biomarkers can serve as non-invasive surrogates for molecular diagnosis, potentially guiding more targeted tissue sampling and molecular testing [[8], [9], [10]].

Previous research has demonstrated that T2-FLAIR mismatch exceeding 42% and nADC values above 2.24 are highly specific for IDH-mutant 1p/19q-intact astrocytomas [9]. In our patient, the posterior lesion exceeded both thresholds (60.6% T2-FLAIR mismatch and nADC of 2.72), which could have prevented its initial misdiagnosis as glioblastoma. Similarly, the minimal T2-FLAIR mismatch (1.8%) and moderate nADC (1.94) of the anterior lesion were more consistent with an IDH-mutant oligodendroglioma than an astrocytoma.

### Implications for tissue sampling and molecular testing

This case underscores the importance of comprehensive molecular profiling of all lesions in multifocal disease, rather than assuming shared molecular features. Initial tissue sampling only assessed 1p/19q status in 1 lesion, leading to a presumptive shared diagnosis that was ultimately incorrect. The nearly 4-year delay in accurate molecular diagnosis may have impacted treatment decisions and patient outcomes. This aligns with recent joint recommendations from Response Assessment in Neuro-Oncology (RANO) groups on standardized tissue sampling, which emphasize sampling multiple regions within tumors and documenting sample locations to correlate with imaging findings [11].

### Differential response to IDH inhibition

Intriguingly, this case provides a unique window into the differential response of distinct IDH-mutant molecular subtypes to targeted therapy within the same patient. The observed reduction in oligodendroglioma volume contrasted with astrocytoma growth during ivosidenib treatment is consistent with findings from the recent INDIGO trial, which demonstrated better response to IDH inhibition in IDH-mutant 1p/19q-codeleted gliomas compared to non-codeleted tumors when treated with vorasidenib [12,13].

This differential response occurred despite non-standard dosing (250 mg twice daily instead of 500 mg twice daily) and reported intermittent usage, suggesting robust activity of IDH inhibition in the oligodendroglioma subtype even with suboptimal dosing. The recent FDA approval of the IDH inhibitor vorasidenib for IDH-mutant gliomas further highlights the clinical relevance of these findings [14].

## Conclusion

This case report documents a rare presentation of concurrent IDH-mutant gliomas with different molecular profiles and highlights the utility of quantitative neuroimaging in accurate non-invasive molecular classification. The observed differential response to IDH inhibitor therapy provides valuable insights into the varying treatment sensitivities of molecular glioma subtypes and reinforces the importance of comprehensive molecular diagnosis in guiding personalized treatment approaches.

The findings suggest that quantitative imaging biomarkers such as T2-FLAIR mismatch percentage and nADC values could serve as valuable adjuncts to histopathology and molecular testing in the diagnostic workflow for gliomas, potentially identifying cases where more extensive molecular profiling is warranted. This case also illustrates the complexity of treating molecularly distinct gliomas and supports emerging evidence of differential sensitivity to IDH inhibition across glioma subtypes, as shown in the recent INDIGO trial [13]. Future research should continue to explore the role of these imaging biomarkers in predicting treatment response to targeted therapies in molecularly distinct glioma subtypes.

## Patient consent

This patient gave written informed consent to be included in our institution’s neuro-oncology research database for data collection and research publication (IRB#10-000655).
